# Establishment of Mouse Teratocarcinomas Stem Cells Line and Screening Genes Responsible for Malignancy

**DOI:** 10.1371/journal.pone.0043955

**Published:** 2012-08-31

**Authors:** Tao Liu, Ying Wang, Xinrong Peng, Liqing Zhang, Jingbo Cheng, Huajun Jin, Mengchao Wu, Qijun Qian

**Affiliations:** 1 Laboratory of Viral and Gene Therapy, Eastern Hepatobiliary Surgical Hospital, The Second Military Medical University, Shanghai, China; 2 Xinyuan Institute of Medicine and Biotechnology, College of Life Science, Zhejiang Sci-Tech University, Hangzhou, Zhejiang, China; Wayne State University School of Medicine, United States of America

## Abstract

The sequential transplantation of embryonal carcinoma cells *in vivo* can accelerate the growth and malignancy of teratocarcinomas. However, the possible molecular mechanisms in this process that reflect cancer formation in the early stage are largely unknown and. To identify which genes are associated with the changes of malignancy of teratocarcinomas, we established a tumorigenesis model in which teratocarcinoma were induced via injecting embryonic stem cells into immuno-deficiency mice, isolating teratocarcinoma stem cell from a teratocarcinoma in serum-free culture medium and injecting teratocarcinoma stem cells into immune-deficient mice continuously. By using high-throughput deep sequence technology, we identified 26 differentially expressed genes related to the changes of characteristics of teratocarcinoma stem cell in which 18 out of 26 genes were down-regulated and 8 genes were up-regulated. Among these genes, several tumor-related genes such as *Gata3*, *Arnt* and *Tdgf1*, epigenetic associated genes such as *PHC1* and *Uty* were identified. Pathway enrichment analysis result revealed that Wnt signaling pathway, primary immunodeficiency pathway, antigen processing and presentation pathway and allograft rejection pathway were involved in the teratocarcinoma tumorigenesis (corrected *p* value<0.05). In summary, our study established a tumorigenesis model and proposed some candidate genes and signaling pathways that may play a key role in the early stage of cancer occurrence.

## Introduction

Teratomas are benign germ cell tumors with somatic tissue or organ components of all three embryonic germ layers. Teratocarcinomas is a kind of malignant teratomas with low spontaneous rate [1] in which malignant stem cells called embryonal carcinoma cells (ECCs) are regarded as equivalent to germ cells or early embryonic cells [2]. In previous studies, a teratocarcinomas model was established via extrauterine transplantation of pregastrulation stage embryos [3], [4], and then Evans et al achieved the establishment of in vitro culture methods of mouse ECCs by co-cultured with fibroblasts [5]. On this basis, isolating mouse embryonic stem cells (ESCs) from blastocysts was achieved [5], [6]. Additionally, homologous mouse or Immuno-deficiency mouse injected with ESCs can also generate teratomas or teratocarcinomas, which became one of a common method to detect the pluripotent of ESCs. However, it also became a security barrier for cell replacement therapy of ESCs.

The study of teratocarcinomas makes cancer “stem cell” or embryonal origin become one of the most widely accepted theory in the process of cancer occurrence. The embryonal rest theory proposed that cancers arose from displacement of embryonic cells and the stem cell theory suggested that tumor arose from dedifferentiation of mature cells or from maturation arrest of immature stem cells [7]. Several researches revealed that ECCs and ESCs give rise to teratocarcinomas in the adult milieu. Also, it can develop into normal individuals when injecting them into blastocysts, indicating that the environmental factors can control the malignant of cells [8], [9], [10]. Another study showed that the sequential transplantation of ECCs in vivo can accelerate the growth and malignancy of teratocarcinomas [11], suggesting that the molecular mechanisms in this process may reflect cancer formation in the early stage.

In our study, we established a tumorigenesis model in which teratocarcinomas were developed via injecting embryonic stem cells into immuno-deficiency mice. We used serum-free and feeder-free 2i culture system [12] to establish ECCs cell lines and sequential transplantation in vivo to obtain higher malignancy level of ECCs cell lines. Furthermore, we performed high-throughput deep sequence technology to identify some malignancy-related genes via comparing the gene expression level between ESCs and series of ECCs which may play a key role in the early stage of cancer occurrence.

## Materials and Methods

### Tumor Induction and Teratocarcinoma Stem Cells Isolation

The Oct4-GFP (Oct-4 promoter driving a green fluorescent protein reporter) ES cell line was present by Professor Qilong Ying [13]. BALB/c athymic mice were purchased from Shanghai Experimental Animal Center, Chinese Academy of Sciences. All animal experiments were carried out in adherence with the National Institutes of Health Guidelines on the Use of Laboratory Animals and approved by the Second Military Medical University Committee on Animal Care (EC11–055). The ES cells were dissociated into a single-cell by trypsin/EDTA and resuspended in phosphate-buffered saline (PBS). About 5×10^6^ ES cells (in 100 microlitres) was injected subcutaneously into inbred BALB/c athymic nude mice. When the diameter of tumor was up to 1.5 cm, the tumor was removed from sacrificed nude mice, washed each three times in PBS and cut into 1cubic millimeter pieces. After digesting by collagenase IV for 30 min at 37°C, the cell suspension was filtered through an 200-mesh sieve and centrifuged for 3 minutes at 100 g. Then the cells were plated in culture flask. The composition of culture medium contained N2B27+2i (Neurobasal 50 ml, DMEM/F12 50 ml, 100×N2 1 ml, 50×B27 2 ml, 100×L-Glutamine 1ml, CHIR99021 3 µM, PD0325901 1 µM). We have successfully established a stable cell line named G1 (Generation I). According to the same method, G1 was inoculated subcutaneously into nude mice, and then teratocarcinomas stem cell named G2 (Generation II) was isolated from the outgrowth tumor. Again, we subsequently repeated the above procedure to obtain cell line named G3 (Generation III). In each generation, a representative teratocarcinomas was took and fixed in 4% paraformaldehyde for 36 hr. After that, teratocarcinomas were embedded in paraffin, then sectioned and stained with hematoxylin and eosin (H&E).

### Limiting Dilution Analysis and Proliferation

For limiting dilution analysis, G1 cells were cultured in 96-well plates in 2i DMEM. Wells with single cell were marked and observed after 6 days. Different generations of teratocarcinoma stem cells and Oct4-GFP ES cells were cultured in 6-well plates at 3×10^5^ cells per well. We passage cells every 3 days, counted cell number, and then calculated doubling time.

### Immunostaining

Cells were prepared for immunostaining via being fixed in 4% paraformaldehyde for 15 min and subsequent permeabilized for 10 min with 0.3% Triton-X in PBS. Cells were then blocked for non-specific binding with 10% normal goat or donkey serum (Abcam) in PBS for 1–2 hr at room temperature. Primary antibodies were diluted in blocking solution and incubated with the samples overnight at 4°C. Samples were rinsed with PBS and incubated with the PE labeled Rabbit Anti-Goat IgG (Invitrogen, 1∶500) for 1 hr at room temperature. Primary antibodies were listed as follows: goat polyclonal IgG Sox2 (sc-54517; Santa Cruz, 1∶100) and goat polyclonal IgG Nanog (sc-30329; Santa Cruz, 1∶100).

### Reverse Transcription PCR

Total RNA was prepared using the RNeasy Mini Kit (Qiagen) with DNaseI treatment. First-strand cDNA was synthesised using Superscript III reverse transcriptase (Invitrogen). The primers for PCR amplification of interested genes were listed in Table S2.

### Comparison of Tumorigenicity

G1, G2, and G3 cells of pair-wise combination were injected subcutaneously into both sides of the nude mice with 5×10^6^ cells. All 12 nude mice in the experiment were killed after 3 weeks. We removed tumor and weighted immediately. Moreover, each representative generation of teratocarcinoma was chosed and fixed in 4% paraformaldehyde for 36 hr. And then fixed teratocarcinoma were embedded in paraffin, sectioned and stained with hematoxylin and eosin (H&E).

### Raw Sequence Reads Preprocessing of Deep Sequence Dataset

RNA sequence was produced by BGI Company via Illumina HiSeq™ 2000 platform. The original image data is transferred into sequence data by base calling, which is named as raw sequence reads and saved as fastq files. Before bioinformatics analysis, data filtering was performed to get the clean reads including three steps: removed reads with adaptors sequence, removed reads in which unknown bases are more than 10% and removed low quality reads which the percentage of the low quality bases of quality value ≤5 is more than 50% in a read. And then the clean reads were mapped to reference sequences using soap2 software [14]. After the preprocessing steps, the gene expression level is calculated by RPKM method (Reads Per kb per Million reads) [15]. The formula of RPKM algorithms is shown as follows:
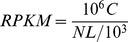



Given RPKM to be the expression of gene A, C represented the number of reads that uniquely aligned to gene A, N represented the total number of reads that uniquely aligned to all genes, and L represented the number of bases on gene A. The RPKM method is able to eliminate the influence of different gene length and sequencing discrepancy when calculating the expression of genes. Therefore, the calculated value can be directly used for comparing the difference of gene expression among experimental samples.

### Bioinformatics Analysis of the Dataset

We performed Fold Change method to identify the genes differed in expression level between three generation samples of teratocarcinoma and ES sample respectively which we called Group1 genes and also identify significant genes among G1, G2 and G3 samples which was named as Group2 genes. Moreover, two-way hierarchical global clustering was performed to all genes using Cluster 3.0 software. We chose centered correlation for array measurement, Euclidean distance for gene measurement and average linkage algorithms.

Functional analysis was performed to map differentially expressed genes in Gene Ontology (GO) database, which is an international standardized gene functional classification system including three ontologies: molecular function, cellular component and biological process. We mapped several interested genes to GO database (http://www.geneontology.org/), and then used hypergeometric test to evaluate significantly enriched GO terms. The p-value calculating formula is:
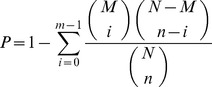



We define N as the number of all genes with GO annotation; n is the number of interested genes in N; M is the number of all genes that are annotated to the certain GO terms; m is the number of interested genes in M.

Furthermore, pathway enrichment analysis was performed to map significant genes in KEGG pathway database via Pathway-Express software. The calculation method of p-value is the same as that of functional analysis. Here N is the number of all genes that with KEGG annotation, n is the number of interested genes in N, M is the number of all genes annotated to specific pathways, and m is number of interested genes in M. A p-value was calculated to each candidate pathway and went through FDR correction. We took corrected p-value ≤0.05 as a threshold. Chromosome analysis was also performed on identified differentially expressed genes. The Gene database in NCBI was used to query the exact chromosome location of interested genes. Chromosome maps were displayed by Endemble.

## Results

### 2i Culture Medium is Suitable for the Growth of Teratocarcinoma Stem Cells

**Figure 1:**
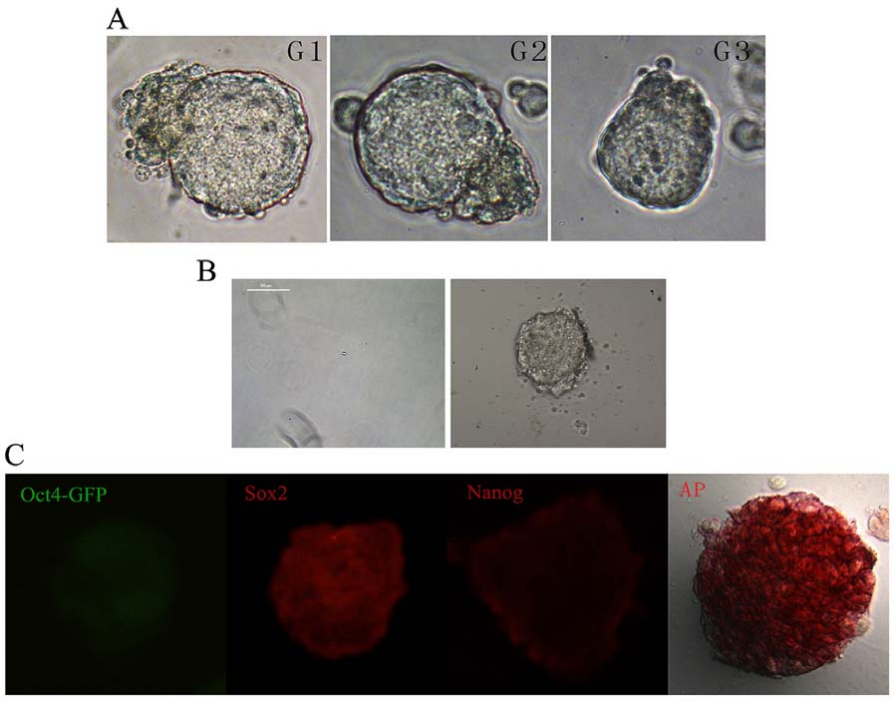
The feature of teratocarcinoma stem cells. A. the phenotype of clones was same as that of ES cells. B. the clones formed by 6-day-cultured single cell. C: expressing pluripotency-related genes including Oct4, Sox2 and Nanog with alkaline phosphatase-positive.

**Figure 2:**
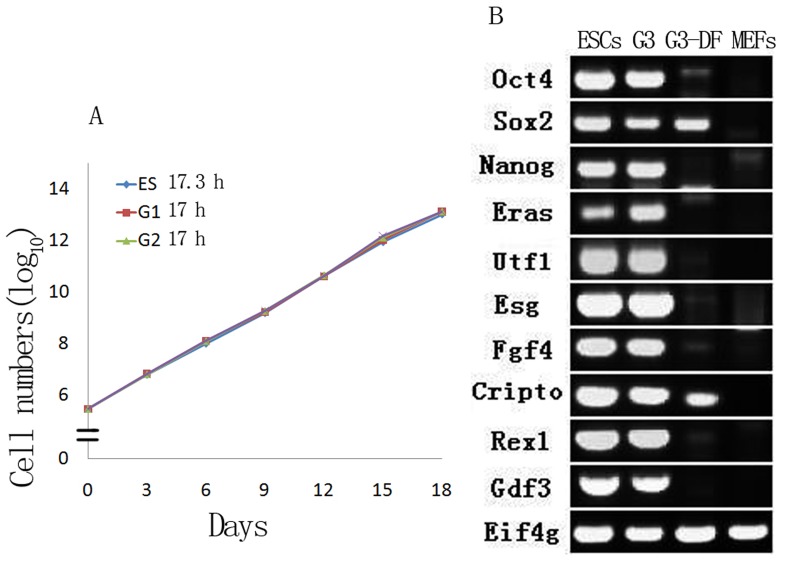
Changes of characteristics of teratocarcinoma stem cells after sequential transplantation. A. the growth curve of teratocarcinoma stem cell marked doubling time. B Decreasing or disappearing expression of pluripotency-related genes of G3 cell cultured by DMEM +10% FBS.

To induce teratocarcinoma, Oct4-GFP ES cells were subcutaneously injected into immuno-deficiency nude mice. Three weeks later teratocarcinoma cells obtained from the tumor were cultured in 2i medium. After 3 days, the compact clones were composed of small cell emerged with ES cell-like phenotype including dome shape, little cytoplasm and high nuclear-cytoplasmic ratio (Figure 1A). The isolated cells were named as G1(Generation I) which can long-term passage in 2i culture condition, maintain ES cells-like phenotype, have rapid proliferation feature and colony formation ability (Figure 1B) and express pluripotent markers of ES cells (Figure 1C). To obtain a second generation of teratocarcinoma, G1 cells were inoculated into nude mice again. We isolated G2 cells using 2i culture system and subsequently obtained G3 cells in the same manner. The characteristics of G2 and G3 cells were the same as those of G1 cells, maintaining ES cell-like phenotype, rapid proliferation and expressing pluripotent markers (Figure 2B).

### Changes of Characteristics of Teratocarcinoma Stem Cells after Sequential Transplantation

First, we compared the proliferation rate among different transplant generation of teratocarcinoma stem cells, identifying that the doubling time of these cells was about the same as those of embryonic stem cells (17 h VS 17.3 h) (Figure 2A). When cultured in DMEM +10% FBS medium, G1 and G2 cells cannot be passage (data not shown). However, G3 cells can grow in a monolayer (denominated G3-DF) and long-term passage (more than 100 passages) but unfortunately lose the majority pluripotent markers of ES cells (Figure S1A, Figure 2B), in which only a few cells showed weakly alkaline phosphatase activity (Figure S1B). Furthermore, when we subcutaneously injected long-term cultured G3-DF cells into immuno-deficiency nude mice, teratocarcinoma outgrew still. It is interesting that if we cultured G3-DF cells long-term cultured with DMEM +10% FBS medium in 2i condition, these cells acquired ES cells-like characteristics again (data not show).

### Increasing Malignant Degree of Teratocarcinoma after Sequential Transplantation

**Figure 3:**
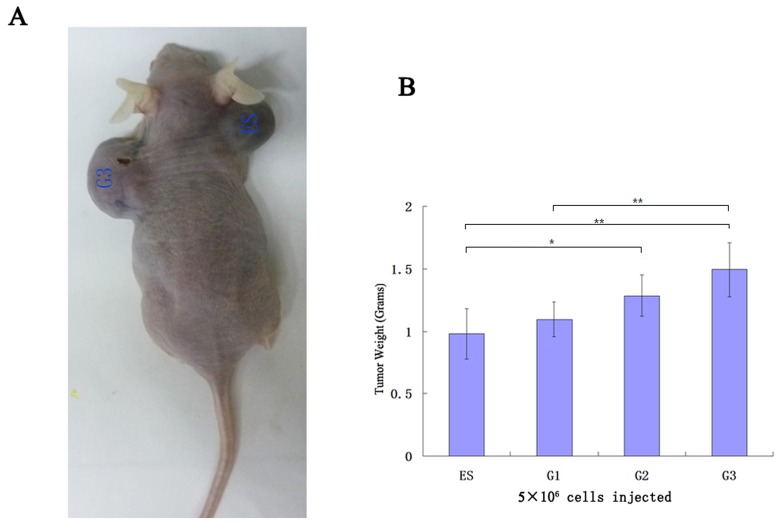
The malignant degree of teratocarcinoma increased after sequential transplantation. A. Different generation of teratocarcinoma stem cells and ES cells were injected subcutaneously into nude mice on both sides of the body; the teratocarcinoma growth of G3 cells was significantly faster than that of ES cells. B. With the increasing of transplantation generation, it accelerated the tumor formation of teratocarcinoma stem cells.

**Figure 4:**
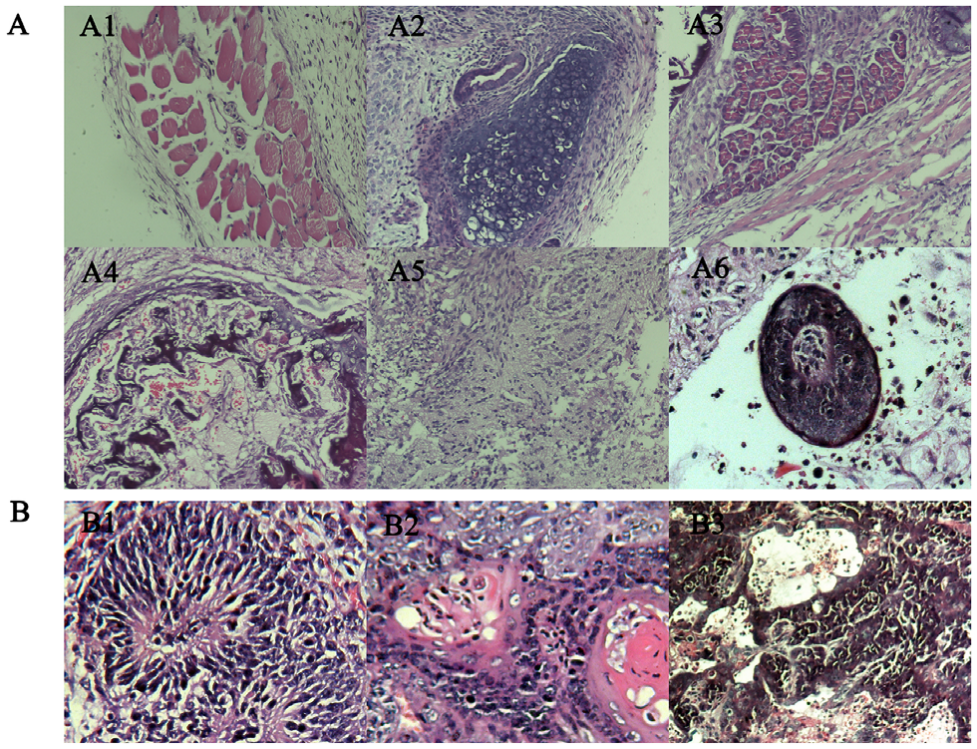
H & E stained tissue sections of teratocarcinoma. A. H & E stained tissue sections of teratocarcinoma formed after inoculating ES cells in nude mice. A1 muscle, A2 cartilage, A3 glands, A4 bone, A5 nerve, A6 hair follicle; B H & E stained tissue sections of teratocarcinoma formed after injecting G3 cells in nude mice. B1 malignant neural tube, B2 squamous cell carcinoma with keratin pearls, B3 adenocarcinoma.

We wonder whether the degree of malignancy increased in vivo correspondingly, for which we compared the growth rate and malignant degree of teratocarcinoma via subcutaneously inoculating different generations of teratocarcinoma stem cells into nude mice on both sides of the body. It showed that after sequential transplantation the tumor formation rate of teratocarcinoma stem cells accelerated in immuno-deficient mice (Figure 3). Teratocarcinoma formed by ES cells mainly composed by a variety of adult tissues containing a small amount of malignant tissue. However, teratocarcinomas formed by sequential transplantation of teratocarcinoma stem cells mainly composed by poorly differentiated adenocarcinoma, carcinomatous nerve tissue and squamous cell (Figure 4). Therefore, we suggested that sequential heterotopic transplantation could accelerate malignant degree of teratocarcinoma stem cells.

### Bioinformatics Analysis of Mouse Teratocarcinoma Deep Sequence Dataset

**Figure 5:**
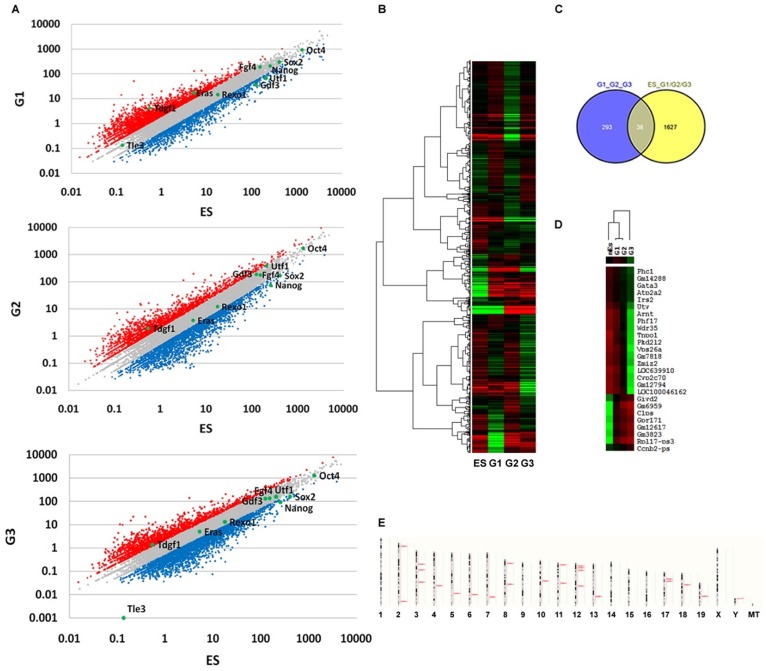
Bioinformatics analysis of mouse teratocarcinoma microarray dataset. A. Scatter plots of global gene expression patterns between teratocarcinoma sample(y axis) and ES sample (x axis). Red, gray and blue represent gene expression levels up, equivalent and down between the samples. The positions of pluripotent genes such as Oct4, Sox2, Nanog were marked by green spot. B. Two-way hierarchical global clustering of microarray dataset C. Overlapping analysis of two groups of genes. D. Two-way hierarchical clustering of 26 identified genes. E. the result of chromosome enrichment analysis of 26 interested genes.

**Figure 6:**
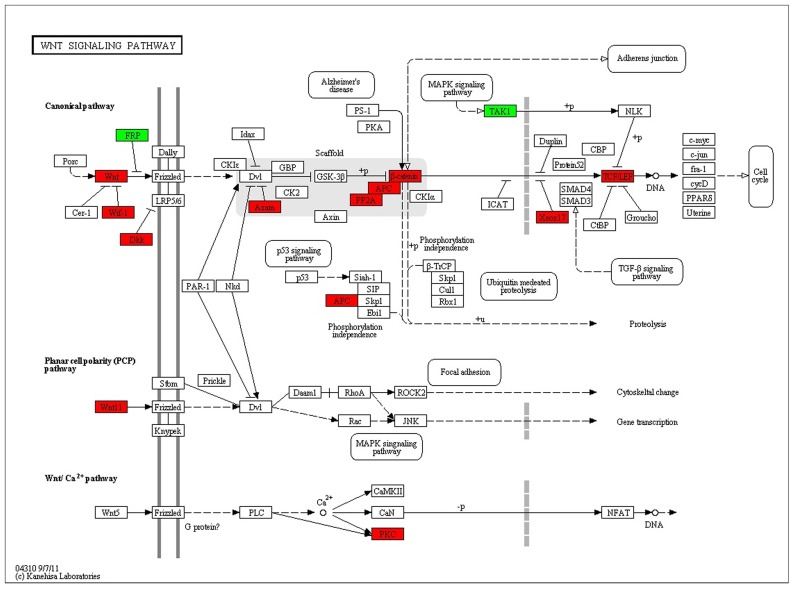
the map of Wnt signaling pathway. The map of wnt signaling pathway included at least three different Wnt pathways: the canonical pathway, the planar cell polarity (PCP) pathway and the Wnt/Ca2+ pathway. Most of the interested genes enriched in canonical pathway. The highly-expressed genes identified from dataset are represented with red color, and the down-regulated genes are represented with green color. The key genes of wnt signaling pathway such as wnt and beta-catenin were identified and marked in the map.

In Table 1, it summarized the detail sample information of RNA sequence dataset via high-throughput deep sequence technology. To evaluate the quality of the sequence raw data, we performed the distribution statistics analysis of all reads (See Figure S2). Figure 5A showed the scatterplots comparing the gene-specific deviation of three generation teratocarcinoma cell line with ES cell lines respectively, in which several pluripotent genes such as Oct4, Sox2, Nanog were marked. Genes with similar expression patterns usually mean functional correlation. Figure 5B described the global cluster analysis of all genes among four samples. When thinking about the differences between ES cell and three teratocarcinoma cell, the 1673 genes whose fold change >2 were up or down regulated consistently among G1 vs. ES, G2 vs. ES and G3 vs. ES samples. These genes were named as Group1 interested genes. Moreover, to investigate the difference among three generations teratocarcinoma samples, we performed the same analysis manner in G1 vs. G2 and G2 vs. G3 samples, 331 genes were satisfied with fold change >2 which we called group2 interested genes. Figure 5C showed the overlapping gene number between group1 and group2, in which 26 of the 38 overlapped genes were up/down expressed consistently among G1 vs. ES, G2 vs. G1 and G3 vs. G2 samples. Figure 5D showed the gene clustering result of these 26 indentified genes. Then, in order to identify the functional classification of these 26 genes, we performed functional enrichment analysis in GeneOntology database. The detail result was listed in Table S1. Furthermore, chromosome enrichment analysis was also performed to these 26 interested genes. In Figure 5E, it showed that chromosome 12 and 3 was enriched by our interested genes which contained four and three genes, respectively. Chromosomes 2,8,11 and 17 enriched two genes. These six chromosomes occupied a total number of 57.7%. In addition, to identify differential genes of Group1/Group2 enriched in which kind of biological pathways, pathway enrichment analysis was performed in KEGG database. The analysis result revealed that wnt signaling pathway, primary immunodeficiency pathway, antigen processing and presentation pathway and allograft rejection pathway were involved in the teratocarcinoma tumorigenesis (corrected p value<0.05). Figure 6 showed the map of Wnt signaling pathway and 17 significant genes were marked on the map in which wnt3a, wnt9a and wnt16 were mapped to the same object (Wnt protein) and Ppp2cb and Ppp2r5 were mapped to PP2A protein.

**Table 1:** The information of mouse RNA sequence dataset.

Sample ID	Sample	Description
1	ES	Mouse embryonic stem cell
2	G1	Mouse teratocarcinoma generation I
3	G2	Mouse teratocarcinoma generation II
4	G3	Mouse teratocarcinoma generation III

## Discussion

Based on the hypothesis that tumor originated in the early stage of remaining embryonic pluripotent cells and the fact that ECCs sequential transplantation in vivo could accelerate the growth and malignancy of teratocarcinomas [11], we obtained teratocarcinoma via injecting ICM origin pluripotent stem cells subcutaneously into immune-deficient mice, and then isolated teratocarcinoma stem cells and used sequential transplantation to simulate early stage of tumor development and progression. Furthermore, we performed microarray analysis to identify candidate genes which may play a key role in the changes of malignancy of teratocarcinoma stem cell.

Teratocarcinoma stem cells have self-renewal and unlimited proliferation feature as same as those of mES cells. Self-renewal is an intrinsic property of mES cells which is not depend on extracellular factors and inhibition of differentiation [12]. Similarly, using small molecule inhibitors (2i) to inhibit the differentiation of teratocarcinoma stem cells can maintain their self-renewal and unlimited proliferation, and with the increasing times of migration, the expression of pluripotency-related genes in teratocarcinoma stem cells and ES cells are considerable which indicated that teratocarcinoma stem cells and mES cells may use the same molecular mechanism to maintain their self-renewal.

Our research is similar to the previous study of using the ECCs [11], after sequential transplantation in vivo the growth and malignancy degree of teratocarcinoma stem cells increased and showed a trend to the neuroectodermal differentiation. And the accelerated formation rate of teratocarcinoma is not due to the increasing growth rate of teratocarcinoma stem cells. The teratocarcinoma stem cell via sequential transplantation can be long-term cultured in DMEM +10% FBS like many other tumor cell lines. In this culture condition, the expression of majority pluripotency-related genes greatly down-regulated or disappear. However, it is also worth to note that the expression of Oct4 is still weak. Several studies indicated that Oct4 played a key role in the generation and maintenance of teratocarcinoma [16]. Other researchers found that Oct4 also expressed in some tumor cells and they speculated that it may be cancer stem cells [17], [18]. In conclusion, these results suggested that the internal changes of teratocarcinoma stem cell are result from sequential transplantation which may reflect the early process of tumor development.

Furthermore, microarray analysis result showed that 26 differentially expressed genes related to the changes of characteristics of teratocarcinoma stem cell in which 18 out of 26 genes were down-regulated and 8 genes were up-regulated. Among these genes, Gata3 and Arnt were well-known tumor-associated genes. Gata3 regulates tumor differentiation, suppresses tumor metastasis in breast cancer [19], [20] and increased Arnt (HIF-1) activity promotes tumor progression. [21] Other genes such as PHC1 and Uty were reported as epigenetic related genes. PHC1 is a component of the Polycomb group (PcG) multiprotein PRC1 complex, a complex required to maintain the transcriptionally repressive state of many genes throughout development including Hox genes. PcG PRC1 complex acts via chromatin remodeling and modification of histones. Uty is a gene that encodes a demethylase. This result is consistent with previous study that indicated epigenetic change played a crucial role in the development of teratoma [22], [23].

Additionally, WNT signaling plays a pivotal role in the tissue lineage differentiation during embryogenesis and the maintenance and self renewal of embryo-derived stem cells in vitro [24]. A lot of researches have proven that tumor stem cell involved many of the same signaling pathways that are found in normal stem cells including Wnt signaling pathways [25], [26], [27], which was thought to play a central role in transformation normal stem cells into cancer stem cells [28]. In this study, pathway enrichment analysis also showed that wnt signaling pathway may play an important function in the process of characteristics changes of teratocarcinoma.

Also, we performed overlapping analysis between our study and Bonner’s [23] study. Among the 53 genes identified by Bonner et al, seven genes, Cxcl1, Gata6, Pdlim4, Rasd1, Tdgf1, Bcat1 and Fst, were overlapped with our analysis. Among these significant candidate genes, the mouse Bcat1 gene has shown to be amplified and over-expressed in a teratocarcinoma cell line [29]. Ben-Yosef’s study indicated that the expression level of Bcat1 is induced during proliferation, the cytosolic BCAT1 activity was detected during fetal development and only in some adult tissues [30]. Previous study also demonstrated that the Gata6 gene regulated Wnt signaling pathway which played a core role in epithelial stem cell development and airway regeneration, and in pancreatic cancer GATA6 activates Wnt signaling [31]. Tdgf1, teratocarcinoma-derived growth factor-1, is also a well-known gene played an essential role in tumor growth and metastasis [32].

In summary, we established a tumorigenesis model in which teratocarcinomas were induced via injecting ES cells into immuno-deficiency mice, then isolated teratocarcinoma stem cells from a teratocarcinoma and injected teratocarcinoma stem cells into immune-deficient mice continuously. Through microarray analysis of teratocarcinoma stem cells, we identified several tumor-related genes, epigenetic associated genes and several significant pathways such as wnt signaling pathway, which may be associated with early onset of tumor development.

## Supporting Information

Figure S1 **Changes of G3 cells cultured in DMEM +10% FBS.** A Monolayer phenotype of G3 cell cultured in DMEM +10% FBS; B Only part of G3 cells keep weakly alkaline phosphatase activity cultured in DMEM +10% FBS.
(JPG)

Figure S2 **the distribution statistics of all reads.** Each figure represented the classification of raw reads in each sample. “Only Adaptor (N1, P1%)” means the number of reads containing adaptor is N1 and the proportion is P1% of total reads. “Cotaining N (N2, P2%)” means the number of reads containing N is N2 and the proportion is P2% of the total reads. “Low Quality (N3, P3%)” means the number of low quality reads is N3 and the proportion is P3% of the total reads. “Clean Reads (N4, P4%)” means the number of clean reads is N4 and the proportion is P4% of the total reads.
(JPG)

Table S1 **the result of GO enrichment analysis of 26 interested genes.**
(DOC)

Table S2 **the primers for reverse transcription PCR.**
(DOC)
