# Supplementary figures and images for: Establishment of Mouse Teratocarcinomas Stem Cells Line and Screening Genes Responsible for Malignancy
Source: PLoS One. 2012 Aug 31;7(8):e43955. doi: 10.1371/journal.pone.0043955 (PMC3432059; doi:10.1371/journal.pone.0043955)

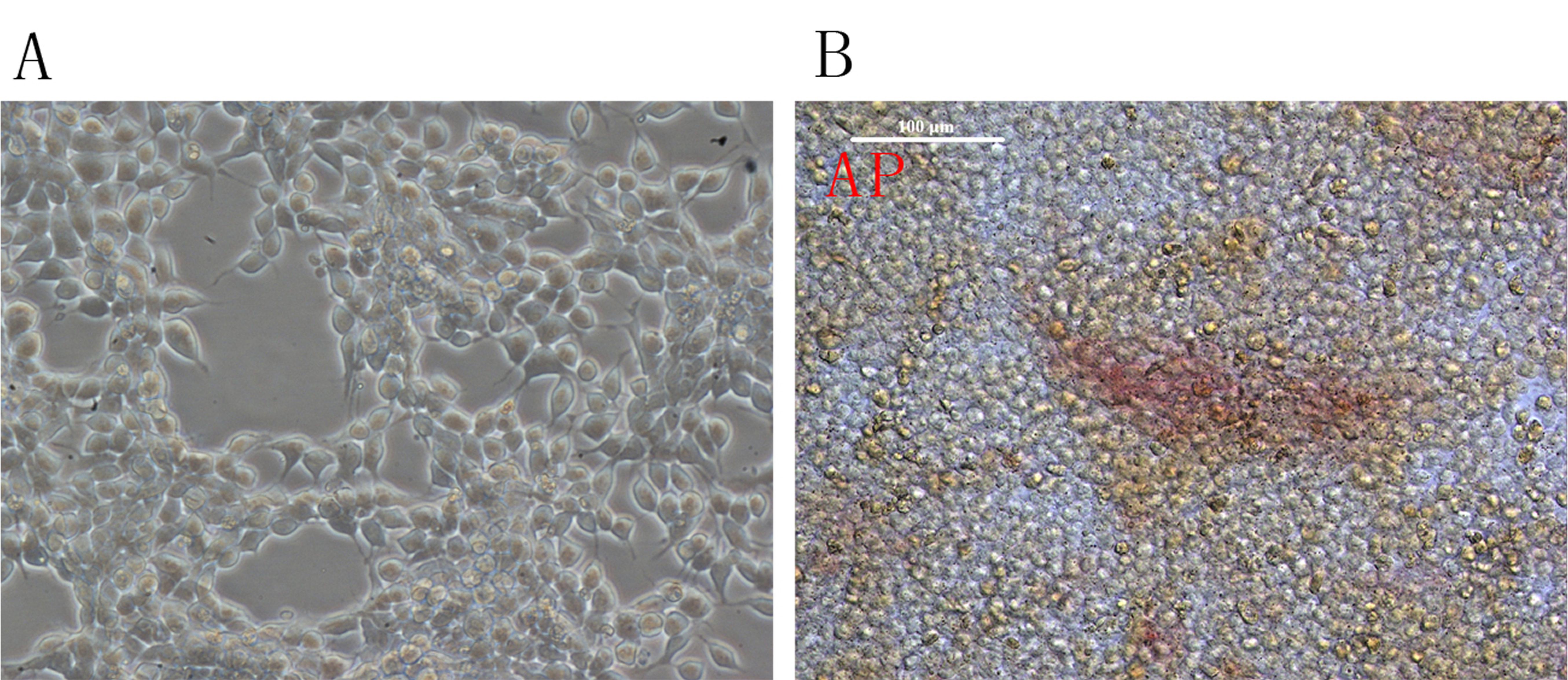

Supplement: Figure S1 — Changes of G3 cells cultured in DMEM +10% FBS. A Monolayer phenotype of G3 cell cultured in DMEM +10% FBS; B Only part of G3 cells keep weakly alkaline phosphatase activity cultured in DMEM +10% FBS. (JPG) [file pone.0043955.s001.jpg]

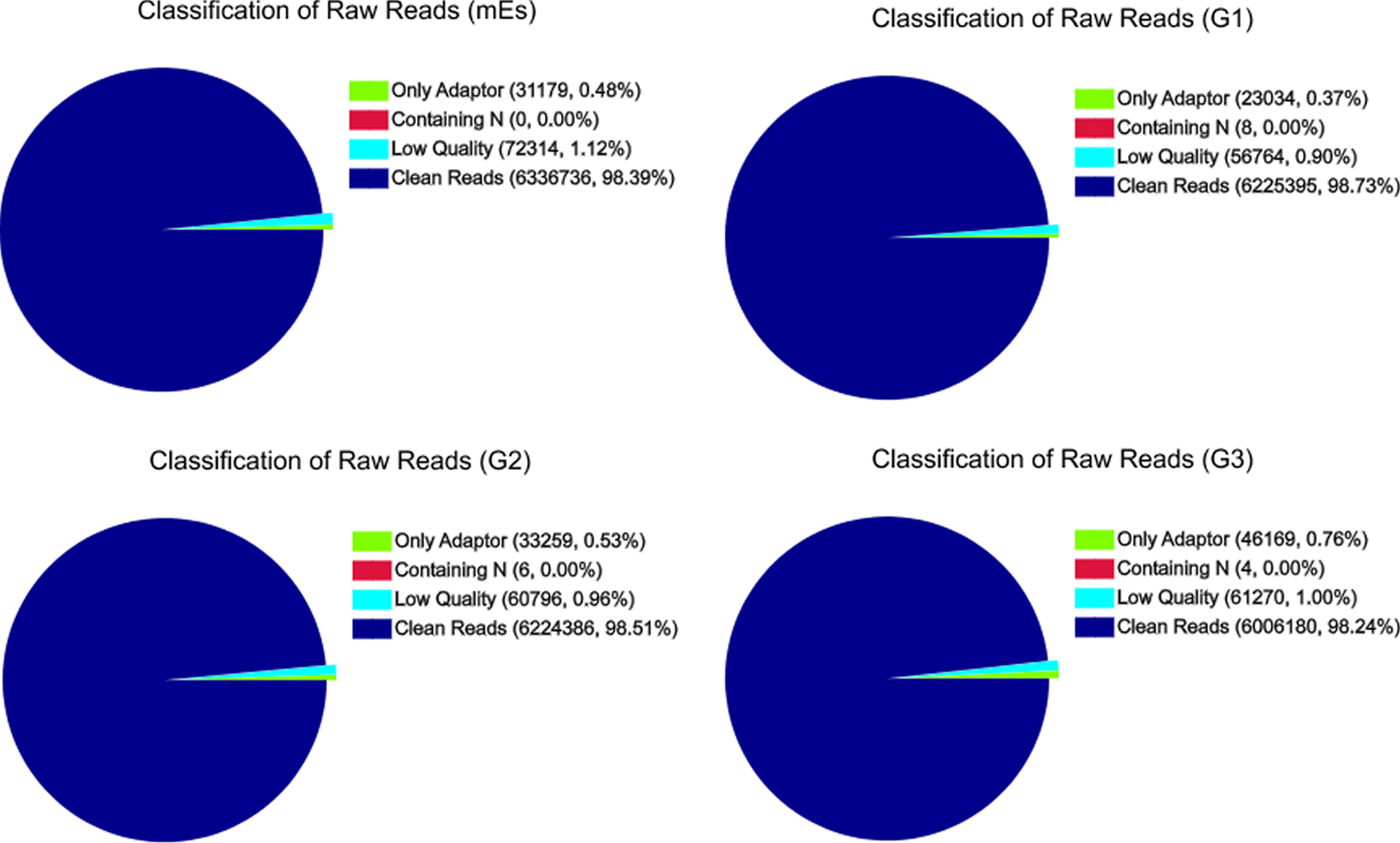

Supplement: Figure S2 — the distribution statistics of all reads. Each figure represented the classification of raw reads in each sample. “Only Adaptor (N1, P1%)” means the number of reads containing adaptor is N1 and the proportion is P1% of total reads. “Cotaining N (N2, P2%)” means the number of reads containing N is N2 and the proportion is P2% of the total reads. “Low Quality (N3, P3%)” means the number of low quality reads is N3 and the proportion is P3% of the total reads. “Clean Reads (N4, P4%)” means the number of clean reads is N4 and the proportion is P4% of the total reads. (JPG) [file pone.0043955.s002.jpg]
