# Supplementary material for: Establishment of Mouse Teratocarcinomas Stem Cells Line and Screening Genes Responsible for Malignancy
Source: PLoS One. 2012 Aug 31;7(8):e43955. doi: 10.1371/journal.pone.0043955 (PMC3432059; doi:10.1371/journal.pone.0043955)
Supplement: Table S1 — the result of GO enrichment analysis of 26 interested genes. (DOC) [file pone.0043955.s003.doc]

**Table S1 the** result of GO enrichment analysis of 26 interested genes

| **Class** | **ID** | **GO function** | **pValue** |
| --- | --- | --- | --- |
| Biological Processes | 1 | positive regulation of hormone metabolic process | 1.778E-04 |
|  | 2 | protein import into nucleus, translocation | 3.113E-04 |
|  | 3 | regulation of hormone metabolic process | 5.085E-04 |
|  | 4 | positive regulation of glucose metabolic process | 8.209E-04 |
|  | 5 | regulation of mesonephros development | 9.050E-04 |
|  | 6 | positive regulation of interleukin-13 secretion | 9.050E-04 |
|  | 7 | fibroblast growth factor receptor signaling pathway involved in ureteric bud formation | 9.050E-04 |
|  | 8 | negative regulation of fibroblast growth factor receptor signaling pathway involved in ureteric bud formation | 9.050E-04 |
|  | 9 | regulation of cell proliferation involved in mesonephros development | 9.050E-04 |
|  | 10 | regulation of glial cell-derived neurotrophic factor receptor signaling pathway involved in ureteric bud formation | 9.050E-04 |
|  | 11 | regulation of cellular response to X-ray | 9.050E-04 |
|  | 12 | negative regulation of cell proliferation involved in mesonephros development | 9.050E-04 |
|  | 13 | regulation of fibroblast growth factor receptor signaling pathway involved in ureteric bud formation | 9.050E-04 |
|  | 14 | negative regulation of glial cell-derived neurotrophic factor receptor signaling pathway involved in ureteric bud formation | 9.050E-04 |
|  | 15 | type IV hypersensitivity | 9.050E-04 |
|  | 16 | positive regulation of interleukin-5 secretion | 9.050E-04 |
| Molecular functions | 1 | lutropin-choriogonadotropic hormone receptor binding | 8.388E-04 |
|  | 2 | metal ion binding | 1.181E-03 |
|  | 3 | cation binding | 1.293E-03 |
|  | 4 | ion binding | 1.324E-03 |
|  | 5 | transcription coactivator activity | 1.365E-03 |
|  | 6 | dioxygenase activity | 2.127E-03 |
|  | 7 | oxidoreductase activity, acting on single donors with incorporation of molecular oxygen, incorporation of two atoms of oxygen | 2.127E-03 |
|  | 8 | oxidoreductase activity, acting on single donors with incorporation of molecular oxygen | 2.178E-03 |
|  | 9 | aryl hydrocarbon receptor binding | 2.514E-03 |
|  | 10 | HMG box domain binding | 2.514E-03 |
|  | 11 | protein transporter activity | 2.611E-03 |
|  | 12 | S100 alpha binding | 4.187E-03 |
|  | 13 | crossover junction endodeoxyribonuclease activity | 5.023E-03 |
|  | 14 | 5'-flap endonuclease activity | 5.858E-03 |
|  | 15 | sequence-specific DNA binding RNA polymerase II transcription factor activity | 6.308E-03 |
|  | 16 | transcription cofactor activity | 6.368E-03 |
|  | 17 | transcription factor binding transcription factor activity | 6.678E-03 |
|  | 18 | RNA polymerase II core promoter proximal region sequence-specific DNA binding transcription factor activity involved in negative regulation of transcription | 6.692E-03 |
|  | 19 | protein binding transcription factor activity | 6.836E-03 |
|  | 20 | core promoter sequence-specific DNA binding | 7.525E-03 |
|  | 21 | endodeoxyribonuclease activity, producing 3'-phosphomonoesters | 7.525E-03 |
|  | 22 | flap endonuclease activity | 7.525E-03 |
|  | 23 | nuclear localization sequence binding | 7.525E-03 |
|  | 24 | calcium-transporting ATPase activity | 7.525E-03 |
|  | 25 | core promoter binding | 8.358E-03 |
| Cellular component | 1 | nuclear chromosome part | 4.973E-05 |
|  | 2 | nuclear chromosome | 9.869E-05 |
|  | 3 | chromosomal part | 1.099E-03 |
|  | 4 | chromosome | 2.076E-03 |
|  | 5 | nuclear lumen | 3.380E-03 |
|  | 6 | retromer complex | 3.546E-03 |
|  | 7 | Slx1-Slx4 complex | 3.546E-03 |
|  | 8 | microsome | 4.646E-03 |
|  | 9 | vesicular fraction | 4.917E-03 |
|  | 10 | nucleoplasm part | 5.618E-03 |
|  | 11 | nuclear chromatin | 6.276E-03 |
|  | 12 | nucleoplasm | 6.851E-03 |
|  | 13 | platelet dense tubular network membrane | 7.080E-03 |
|  | 14 | nuclear part | 7.430E-03 |
|  | 15 | platelet dense tubular network | 7.961E-03 |
|  | 16 | intracellular organelle lumen | 8.764E-03 |
|  | 17 | organelle lumen | 9.558E-03 |
