# Supplementary material for: Establishment of Mouse Teratocarcinomas Stem Cells Line and Screening Genes Responsible for Malignancy
Source: PLoS One. 2012 Aug 31;7(8):e43955. doi: 10.1371/journal.pone.0043955 (PMC3432059; doi:10.1371/journal.pone.0043955)
Supplement: Table S2 — the primers for reverse transcription PCR. (DOC) [file pone.0043955.s004.doc]

**Table S2 the primers for reverse transcription PCR**

| **Genes** | **Primers** |
| --- | --- |
| Eras | F- ACTGCCCCTCATCAGACTGCTACT, R- CACTGCCTTGTACTCGGGTAGCTG; |
| Esg1 | F- GAAGTCTGGTTCCTTGGCAGGATG, R- ACTCGATACACTGGCCTAGC; |
| Fgf4 | F- CGTGGTGAGCATCTTCGGAGTGG, R-CCTTCTTGGTCCGCCCGTTCTTA; |
| Utf1 | F-GGATGTCCCGGTGACTACGTCTG, R- GGCGGATCTGGTTATCGAAGGGT; |
| Cripto | F-ATGGACGCAACTGTGAACATGATGTTCGCA,R-CTTTGAGGTCCTGGTCCATCACGTGACCAT; |
| Rex1 | F- ACGAGTGGCAGTTTCTTCTTGGGA, R-TATGACTCACTTCCAGGGGGCACT; |
| Gdf3 | F- GTTCCAACCTGTGCCTCGCGTCTT, R-AGCGAGGCATGGAGAGAGCGGAGCAG; |
| Oct4 | F- TCTTTCCACCAGGCCCCCGGCTC, R-TGCGGGCGGACATGGGGAGATCC; |
| Sox2 | F- TAGAGCTAGACTCCGGGCGATGA, R- TTGCCTTAAACAAGACCACGAAA; |
| Nanog | F- CAGGTGTTTGAGGGTAGCTC, R- CGGTTCATCATGGTACAGTC; |
| Eif4g2 | F-ATTCTTCGTTGTCAAGCCGCCAAAGTGGAG,R-AGTTGTTTGCTGCGGAGTTGTCATCTCGTC |
